# The Emerging Role of Osteocytes in Cancer in Bone

**DOI:** 10.1002/jbm4.10186

**Published:** 2019-02-27

**Authors:** Emily G Atkinson, Jesús Delgado‐Calle

**Affiliations:** ^1^ Department of Anatomy and Cell Biology Indiana University School of Medicine Indianapolis IN USA; ^2^ Department of Medicine Division of Hematology/Oncology Indiana University School of Medicine Indianapolis IN USA; ^3^ Indiana Center for Musculoskeletal Health Indiana University School of Medicine Indianapolis IN USA

**Keywords:** OSTEOCYTES, MYELOMA, BONE RESORPTION, BONE FORMATION, CANCER

## Abstract

Advances in the last decade have established the osteocyte, the most abundant cell in bone, as a dynamic and multifunctional cell capable of controlling bone homeostasis by regulating the function of both osteoblasts and osteoclasts. In addition, accumulating evidence demonstrates that osteocyte function is altered in several skeletal disorders, and targeting osteocytes and their derived factors improves skeletal health. Despite the remarkable progress in our understanding of osteocyte biology, there has been a paucity of information regarding the role of osteocytes in the progression of cancer in bone. Exciting, recent discoveries suggest that tumor cells communicate with osteocytes to generate a microenvironment that supports the growth and survival of cancer cells and stimulates bone destruction. This review features these novel findings and discussions regarding the impact of chemotherapy on osteocyte function and the potential of targeting osteocytes for the treatment of cancer in bone. © 2019 The Authors. *JBMR Plus* published by Wiley Periodicals, Inc. on behalf of American Society for Bone and Mineral Research.

## Introduction

The skeleton is a multifunctional tissue that provides support and protection to various organs of the body, regulates mineral homeostasis and hematopoiesis, enables body movement, and has multiple endocrine functions in the body. Bones are composed of a calcified extracellular matrix and a multitude of cells that establish complex interactions to maintain bone homeostasis. Osteoclasts derive from hematopoietic precursors and are responsible for bone resorption, a process that breaks down bone into its mineral and collagenous constituents. Cells of the osteoblastic lineage derive from mesenchymal stem cells, a multipotent cell population with capacity to differentiate into osteoblasts, osteocytes, adipocytes, chondrocytes, and myoblasts.^1^, ^2^ The main function of osteoblasts is bone formation. Osteoblasts secrete a variety of proteins that constitute the bone matrix and become mineralized. Upon completing bone formation, a fraction of osteoblasts becomes entombed by mineralized matrix and differentiates into osteocytes. Osteocytes are the most abundant cells in bone and considered permanent residents of skeletal tissue, with an estimated half‐life of 25 years;^3^, ^4^ however, the life of many osteocytes may be shorter.^5^, ^6^ Although initially described as passive cells, we now know that osteocytes are multifunctional cells that sense and transduce mechanical forces in bone, and coordinate both bone formation and bone resorption by secreting cytokines that control the activity of osteoblasts and osteoclasts (reviewed in Delgado‐Calle and Bellido^7^ and Bonewald^8^).

As occurs in other organs in the body, turnover of cells and matrix also takes place in bone and is essential to maintain tissue integrity. Through a complex and tightly regulated process known as “bone remodeling,” old or damaged bone is removed by osteoclasts and subsequently replaced by new bone formed by osteoblasts.^9^ Under physiological conditions, bone remodeling occurs in compartmentalized structures known as “bone remodeling units,” which enable bone resorption and bone formation to occur in a balanced and sequential manner at the same anatomical location.^10^, ^11^, ^12^, ^13^ Alteration of osteoblasts and osteoclasts activities within these remodeling units leads to the development of bone disorders. Imbalance in favor of resorption results in bone loss and a deterioration of bone microarchitecture, whereas elevation of bone formation is usually associated with increased bone mass.

Different kinds of cancer cells can grow in bone. Primary bone tumors are rare and account for a small portion of newly diagnosed cancers. These bone tumors arise from cells present in the bone tissue and include osteosarcomas, which typically occur in adolescents and are thought to arise from osteoblasts;^14^ chondrosarcomas, which begin in cartilage and are more frequent in adults; and Ewing sarcomas and chordomas. Other cancers begin in bone but do not arise from bone cells. For instance, multiple myeloma is a cancer of plasma cells that originates in the bone marrow and causes bone tumors and bone lesions in 80% of myeloma patients.^15^, ^16^ Lastly, metastatic bone tumors develop from cancer cells that originated in another area of the body and then migrate and spread to the bone. Bone metastases are more common than primary bone cancers in adults. In the majority of patients, the primary tumor is located in the prostate or the breast, which account for 70% of skeletal metastases (reviewed in Macedo and colleagues^17^). Bone metastases are frequently one of the first signs of disseminated disease in cancer patients and typically indicate a short‐term prognosis. The growth of cancer cells in bone has a deleterious impact on patients’ quality of life and represents a significant cause of morbidity and mortality.^18^, ^19^, ^20^ Patients with bone tumors frequently present with severe pain, impaired mobility, spinal cord compression, pathologic fractures, bone marrow aplasia, and hypercalcemia.

Autopsy observations made in women with breast cancer led Paget to propose the “seed and soil” hypothesis in which the bone (soil) supports the growth of the breast cancer cells (seed).^21^ Later, work by Mundy^22^ and by TJ Martin and colleagues^23^ showed that indeed cancer cells establish interactions with osteoblasts and osteoclasts present in the bone/bone marrow compartment leading to a “vicious cycle” that alters bone homeostasis and fuels tumor growth (recently reviewed in Croucher and colleagues^24^). The growth of cancer cells in the bone/bone marrow microenvironment alters normal bone remodeling, thus leading to the development of bone disease. In cancer‐induced osteolytic bone disease, as occurs in breast cancer metastasis and multiple myeloma, tumor cells stimulate osteoclastogenesis and bone resorption, primarily in a parathyroid‐related protein (PTHrP)/receptor activator of nuclear factor kappa‐Β ligand (RANKL)‐mediated manner. Increased bone resorption releases bone matrix–embedded growth factors such as transforming growth factor beta (TGF‐β),^25^, ^26^ which further stimulates tumor growth and bone destruction.^27^ Concomitantly, cancer cells secrete factors (interleukin [IL]‐7, IL‐3, Dickkopf Wnt Signaling Pathway Inhibitor 1 [DKK‐1], and Sclerostin) and promote epigenetic changes that suppress osteoblast differentiation and function (reviewed in Giuliani and colleagues,^(28)^ MacDonald and Delgado‐Calle,^29^ and Adamik and colleagues^30^). As a result, bone is resorbed at a rate faster than it is formed, causing the development of overt “osteolytic lesions,” which severely weakens the bone and elevates fracture risk. In osteoblastic lesions, as occurs in osteosarcomas and prostate cancer metastasis, tumor‐derived factors, including insulin‐like growth factor (IGF)‐1 and −2, TGF‐β, bone morphogenetic proteins (BMPs), platelet‐derived growth factor (PDGF), endothelin‐1 (ET‐1), and fibroblast growth factors (FGFs) stimulate the differentiation and bone‐forming activity of osteoblasts (reviewed in Guise and Mundy^31^). However, the bone produced is formed primarily of woven tissue and exhibits compromised mechanical properties. In turn, osteoblasts produce growth factors such as IL‐6, monocyte chemoattractant protein 1 (MCP‐1), or vascular endothelial growth factor (VEGF) that further stimulate tumor growth.^31^ More recent studies revealed that cancer cells also interact with other cells in the bone/bone marrow microenvironment, including osteocytes, adipocytes, endothelial cells, and immune cells. In particular, growing evidence indicates that osteocytes are important contributors to tumor progression in bone and the associated skeletal disease.^29^, ^32^, ^33^ This review summarizes the current knowledge of the role of osteocytes in the progression of cancer in bone, the mechanisms by which osteocytes communicate with cancer cells, and the potential of targeting osteocytes for the treatment of cancer‐induced bone disease.

## Role of Osteocytes in Bone Physiology

Osteocytes live deep within mineralized bone in small pockets known as lacunae. Osteocytes have long cytoplasmic processes that run through small channels called canaliculi, establishing a complex and extensive canalicular network in the mineralized bone.^2^, ^8^ These processes reach periosteal and endocortical surfaces of cortical bone, as well as the bone marrow surface. Through this lacunar‐canalicular system, osteocytes connect among themselves, establish cell‐to‐cell interactions with cells on the bone surfaces, and distribute autocrine/paracrine secreted factors.

Work performed over the last decade has brought about a revolution of our understanding of the role of osteocytes in bone biology. Osteocytes regulate bone formation by controlling osteoblast differentiation, survival, and function. Earlier work showed that osteocytes are the main mechanosensors in the skeleton by sensing mechanical forces and translating them into biochemical signals that promote bone formation.^34^, ^35^ Supporting this notion, targeted deletion of osteocytes impairs the bone anabolic response to mechanical loading.^36^ Some of the molecular cues by which osteocytes regulate bone formation have been identified in more recent studies. Extensive clinical and animal data demonstrated that osteocytes negatively regulate osteoblast viability and function by secreting Wnt signaling antagonists, including DKK‐1 and Sclerostin (reviewed in Delgado‐Calle and Bellido,^7^ Delgado‐Calle and colleagues,^37^ and Baron and Kneissel^38^). Both DKK‐1 and Sclerostin inhibit Wnt signaling by blocking the binding of Wnt ligands to Frizzled receptors and low‐density lipoprotein receptor‐related proteins (LRP) 5 and 6.^39^, ^40^ Patients with mutations in DKK‐1 and SOST, the gene encoding Sclerostin, as well as mice with genetic deletion of these genes, exhibit increased bone mass, mainly due to elevated osteoblast number and bone formation. These initial observations attracted considerable attention to DKK‐1 and Sclerostin and the role of the Wnt signaling pathway in bone. Extensive work in this area showed that osteocytes coordinate the osteogenic response to mechanical forces by downregulating Sclerostin, thus enabling activation of canonical Wnt signaling.^37^, ^39^, ^40^, ^41^, ^42^ Sclerostin is also downregulated by parathyroid hormone (PTH), an FDA approved agent used in the clinic to stimulate bone formation in osteoporotic patients.^43^, ^44^, ^45^, ^46^, ^47^ Several neutralizing antibodies against DKK‐1, Sclerostin, and other components of the Wnt signaling pathway (i.e., LRP4) have been developed and have shown promising therapeutic outcomes for patients with osteoporosis and other skeletal diseases (discussed in the section “osteocytes and their derived factors as targets for the treatment of cancer that grows in bone”). However, the cell orchestrating the anabolic actions of the Wnt signaling pathway in bone has remained elusive. One hypothesis is that osteocytes coordinate the anabolic actions of canonical Wnt/β‐catenin signaling activation in bone. Consistent with this notion, genetic stimulation of Wnt/β‐catenin signaling in osteocytes results in increased osteoblast number and bone formation, leading to net bone gain.^48^ These results contrast with those observed in mice expressing the same dominant active β‐catenin transgene in osteoblasts, which also exhibit bone gain, but mainly due to decreases in bone resorption.^49^

Osteocytes also produce a number of cytokines controlling the differentiation and function of osteoclasts. The high bone mass displayed by mice lacking RANKL in osteocytes supports that osteocytes are a major source of RANKL during adult bone remodeling bone.^50^, ^51^ Osteoprotegerin (OPG) expression, which competes with RANKL for binding to receptor activator of nuclear factor κ (RANK) in osteoclast precursors, is regulated by the Wnt/β‐catenin pathway, and mice lacking β‐catenin in osteocytes have increased osteoclast number and bone resorption.^52^ Moreover, osteocytes are an additional source of secreted macrophage colony‐stimulating factor (M‐CSF) in bone.^53^ An area of intense investigation is the role of apoptotic osteocytes in the regulation of local bone resorption. It has been shown that decreases in osteocyte life span accompany the increased bone resorption and bone loss associated with estrogen and androgen deficiency, glucocorticoid excess, mechanical disuse, and aging.^54^, ^55^, ^56^ Further, osteocyte apoptosis is spatially, temporally, and functionally linked to the removal and replacement of damaged bone. Mechanistically, osteocyte apoptosis increases the expression of RANKL in neighboring osteocytes, which in turn recruits osteoclast precursors and stimulates their differentiation.^57^, ^58^, ^59^ Consistent with this notion, pharmacologic inhibition of apoptosis impairs the increase in RANKL expression induced by unloading or overloading.^57^, ^58^ In contrast, inhibiting osteocyte apoptosis with a bisphosphonate that targets osteocytes and osteoblasts also prevented the increase in RANKL but did not prevent the bone loss induced by unloading,^59^ indicating that apoptotic osteocytes can also regulate osteoclast precursor recruitment to specific areas in a RANKL‐independent manner (ie, OPG, high mobility group box 1 [HMGB1], tumor necrosis factor [TNF], IL‐6, and VEGF).^60^, ^61^

## Role of Osteocytes in Tumor Metastasis to Bone

Metastasis of cancer cells to a secondary site is a multistep process involving detachment from the primary tumor and egress to the bloodstream.^24^, ^62^ Tumor cells circulate and leave the bloodstream to finally colonize the bone, homing to specific niches within the bone marrow.^24^, ^62^ It is becoming evident that cancer cell colonization and homing to bone is not a casual event but rather regulated by the existence of “premetastatic” niches that prime the arrival of metastatic cells to areas with favorable conditions for cancer cell survival. However, the specific mechanisms by which cancer cells localize and arrive to these areas remain to be determined. Similarly, the role of osteocytes in the early steps of tumor metastasis and homing to bone is unclear. Genetic ablation of osteocytes, induced by diphtheria toxin administration, increased the homing of myeloma cells to particular areas of the bone and increased total tumor burden.^63^ Mechanistic studies showed that the expression of IL‐6, hepatocyte growth factor (HGF), hypoxia‐inducible factor (HIF)‐1, cytokines known to be involved in myeloma migration and homing to the bone marrow, as well as immune‐suppressive cell populations (myeloid‐derived suppressor cells, regulatory T and B cells), were elevated in the bone marrow of mice with apoptotic osteocytes. In addition, it has been shown that C‐X‐C motif chemokine ligand 12 (CXCL12) signaling through the C‐X‐C chemokine receptor type 4 (CXCR4) receptor in cancer cells plays an important role in the retention and homing to bone of both cancers that develop in the bone marrow, as well as metastatic cancer cells.^64^, ^65^, ^66^ Osteocytes produce CXCL12 and therefore could activate the CXCL12‐CXCR4 signaling axis in cancer cells, favoring their homing to bone.^67^ However, alternatively it is also possible that osteocytes prevent the migration and arrival to bone of metastatic breast cancer cells. In vitro studies show that mechanically stimulated osteocytes reduced the transendothelial migration of breast cancer cells.^68^ Further, interactions between osteocytes and endothelial cells decrease the expression of matrix metalloproteinase 9 (MMP‐9), an enzyme known to facilitate the movement of metastatic cancer cells through the extracellular matrix.^68^ The disparity between these findings suggest that interactions between osteocytes and tumor cells are context and cancer specific. In fact, osteocytes have distinct effects in the migratory potential of breast and prostate cancer cells.^69^

Although osteocytes have the potential to participate in the establishment of bone metastasis and homing to specific areas of the bone, their role in this process remains largely unknown. Future research efforts are required to determine the specific contribution of osteocytes to the relocation and homing of cancer cells to bone. Identification of the cellular and molecular events that mediate the cross‐talk between cancer cells and bone cells should help to identify therapeutic targets that can interfere with the first steps of bone metastasis.

## Role of Osteocytes in Cancer Cell Dormancy and Tumor Proliferation in Bone

Once cancer cells arrive to bone, most of them undergo apoptosis with only a few becoming dormant and surviving. Eventually, when better conditions for growth are established, dormant cells are activated and proliferate, thus initiating the bone destruction process.^24^, ^70^ Because dormant cells are resistant to chemotherapies and can repopulate the tumor after treatment cessation, it is critical to understand the mechanisms controlling dormancy to either maintain dormant cells or awaken them after reestablishing sensitivity to chemotherapy. The specific mechanisms regulating tumor cell dormancy and reactivation in bone are just starting to be revealed. New data indicate that osteoblasts and osteoclasts are important players in the regulation of dormancy. In vitro and in vivo experiments have shown that upon arrival to bone, cancer cells interact with osteoblasts on the endosteal surface, which in turn maintains cancer cells in a dormant state by inhibiting their proliferation.^24^, ^71^, ^72^, ^73^ In contrast, induction of osteoclast differentiation and bone resorption decreases the number of dormant cancer cells present in the endosteal niche, suggesting that osteoclasts control the reactivation of dormant cells.^24^, ^71^, ^74^, ^75^, ^76^ As of today, there is no experimental data linking osteocytes to the regulation of cancer cell dormancy. However, given the critical role of osteocytes in bone remodeling, it is possible that they regulate dormancy through indirect actions on osteoblasts and osteoclasts. Future studies are warranted to determine the role of osteocytes in cancer cell dormancy.

After reactivation, cancer cells establish multiple interactions with cells in the bone/bone marrow microenvironment that favor a continued stimulation of tumor growth. As discussed earlier, tumor cells initiate a “vicious cycle” in which cancer cells produce and stimulate other cells to secrete factors that increase osteoclast resorption. In turn, bone resorption releases growth factors from the bone matrix that promote tumor proliferation and survival.^77^, ^78^ Although originally not included in this “vicious cycle,” new findings support that osteocytes also contribute to the generation of a microenvironment that is conducive to tumor proliferation through both direct and indirect mechanisms. Osteocytes are the main source of RANKL in adult bone. In addition, the prevalence of osteocytes expressing RANKL is increased in bones involved with myeloma cells (see the section “osteocytes and their derived factors as targets for the treatment of cancer that grows in bone”).^79^ Furthermore, myeloma cells increase the expression of IL‐11 in osteocytes, a cytokine that favors osteoclast differentiation.^80^ Thus, it is possible that by secreting pro‐osteoclastogenic cytokines and stimulating bone resorption, osteocytes contribute to the release of growth factors from the matrix fueling tumor growth.

**Figure 1:**
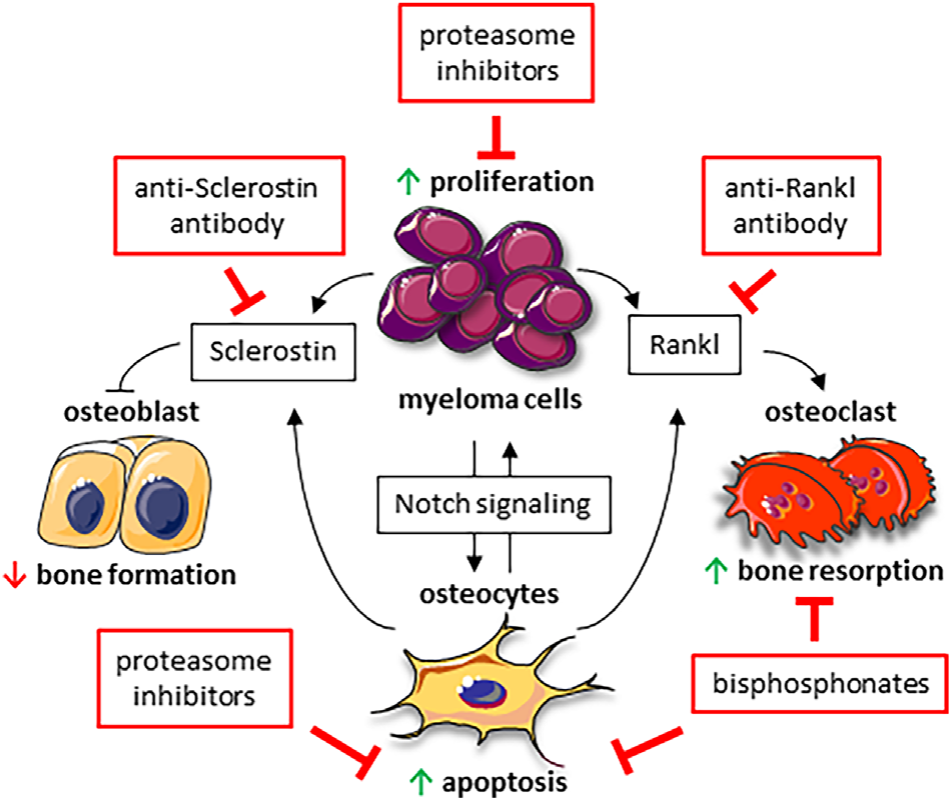
Communication between cancer cells and osteocytes contributes to tumor progression and bone destruction. Cell‐to‐cell communication between osteocytes and myeloma cells results in bidirectional Notch signaling activation, leading to osteocyte apoptosis and stimulation of myeloma cell proliferation. In addition, osteocyte apoptosis leads to increased RANKL expression, which acts as a chemoattractant to recruit osteoclast precursors to local areas and promote their differentiation, thus initiating bone resorption. In addition, myeloma cells increase SOST/Sclerostin production by osteocytes, which in turn decreases Wnt signaling and inhibits osteoblast differentiation and function. Recent findings support that the beneficial effects of bisphosphonates and proteasome inhibitors in cancer patients are also due to the prevention of osteocyte (and osteoblast) apoptosis. In addition, preclinical data have shown that targeting the osteocyte‐derived factor Sclerostin stimulates osteoblast differentiation and new bone formation in bones infiltrated with cancer cells. Similarly, pharmacologic inhibition of RANKL, abundantly expressed by osteocytes in adult bone, prevents skeletal‐related events in cancer patients.

Osteocytes also regulate tumor proliferation by direct interactions with cancer cells (Fig. 1). Osteocytes establish physical interactions with myeloma cancer cells located in the endocortical surface of the bone.^79^ These physical interactions result in the activation of Notch signaling in myeloma cells, a pathway that mediates cell‐to‐cell communication between neighboring cells and controls proliferation/death programs.^79^ Activation of Notch signaling by osteocytes increases cyclin D1 mRNA expression and accelerates the proliferation of myeloma cells.^79^ In vitro, pharmacological inhibition of Notch signaling using gamma‐secretase inhibitors (GSIs) fully prevents the increase in myeloma proliferation induced by osteocytes.^79^ Because dysregulation of Notch signaling contributes to the progression of several cancers in bone,^81^, ^82^ this pathway provides a therapeutic opportunity to treat cancers in bone. However, Notch signaling plays critical roles in several organs, and systemic inhibition of Notch has several side effects that limit its use in the clinic.^82^, ^83^ Thus, it is important to identify the specific Notch components (receptors and ligands) involved in the interactions between cancer cells and bone cells as this is key for the development of safer therapies. In this regard, both autocrine and paracrine (with osteoblasts) activation of Notch receptor 1 and 2 signaling stimulate the proliferation of myeloma cells.^84^, ^85^, ^86^, ^87^ Interestingly, osteocytes could use a different set of Notch receptors to communicate with myeloma cells, as bidirectional communication between osteocytes and myeloma cells changed the Notch receptor repertoire in both cell types, with a rapid and marked increase in the expression of Notch receptor 3 and Notch receptor 4.^79^

Osteocytes also contribute to the growth of metastatic cancers in bone. For instance, Keller and colleagues demonstrated in vivo that the growth of prostate cancer cells in bone increases intramedullary pressure.^88^ Because osteocytes are the main mechanotransducers in bone, they investigated whether these changes in physical forces in bone alter osteocyte function. In vitro, conditioned media from pressurized osteocytes increased the proliferation, migration, and invasion capacity of prostate cancer cells.^88^ Mechanistic studies revealed that these effects were partially mediated by the osteocyte‐derived C‐C motif chemokine ligand 5 (CCL5) and matrix metalloproteinases.^88^

In concert, these findings identify osteocytes as important pro‐tumorigenic cells. However, because most of the data currently available are from in vitro and ex vivo co‐culture systems, future studies are required to assess the specific contribution of osteocytes to tumor growth in vivo.

## Osteocytes and Dysregulation of Bone Remodeling in Bone Colonized by Cancer Cells

The growth of cancer cells in bone alters the function of osteoblasts and osteoclasts, resulting in profound alterations of bone remodeling that ultimately compromised bone integrity. We now have confirmation that osteocytes actively participate in the development of osteolytic lesions (Fig. 1). Experiments performed in the frame of multiple myeloma have shown that the bone remodeling compartment is disrupted by myeloma cells,^89^ allowing myeloma cells to interact with other cells in the bone marrow, including osteocytes. It is well accepted that myeloma cells stimulate bone resorption through the production of several pro‐osteoclastogenic factors and via interactions with osteoblasts (reviewed in Silbermann and Roodman^20^ and in Roodman^90^, ^91^). However, in vitro and in vivo work demonstrates that myeloma cells also increase the expression of RANKL in osteocytes.^79^ In addition, the expression of the Wnt target gene OPG is decreased in osteocytes exposed to myeloma cells, thus increasing even further the RANKL/OPG ratio and the osteoclastogenic potential of osteocytes.^79^ Myeloma cells also increase the expression of other pro‐osteoclastogenic cytokines in osteocytes, such as interleukin (IL)‐11.^80^ In addition, myeloma cells decrease the life span of osteocytes in bone, as occurs in other bone disorders characterized by bone loss (see the section “role of osteocytes in bone physiology”). Myeloma patients exhibit more apoptotic osteocytes in bone than healthy subjects or patients with monoclonal gammopathy of undetermined significance (MGUS), a condition with abnormal paraprotein serum levels but absence of the typical multiple myeloma symptoms.^80^ Similarly, in preclinical models of multiple myeloma bone disease, osteocyte apoptosis is increased in bone areas infiltrated with myeloma cells.^79^ Mechanistic studies demonstrated that myeloma cells induce osteocyte apoptosis via physical interactions with osteocytes. Cell‐to‐cell communication between myeloma cells and osteocytes activate Notch signaling in osteocytes, triggering caspase‐3 mediated apoptosis.^79^ In addition, TNFα secreted by myeloma cells sustains/amplifies osteocyte apoptosis.^79^ Moreover, Giuliani and colleagues showed that myeloma cells also stimulate osteocyte apoptosis by inducing autophagy.^92^ Consistent with the notion that apoptotic osteocytes attract osteoclast precursors to initiate targeted local bone resorption, conditioned media from osteocytes exposed to myeloma cells stimulates the recruitment of osteoclast precursors, and this effect was fully prevented by an inhibitor of osteocyte apoptosis.^79^ Together, these results identify apoptotic osteocytes as important contributors to the exaggerated bone resorption that drives the development of cancer‐induced osteolytic lesions.

In addition to increased osteoclast number, myeloma‐induced bone lesions also exhibit reduced osteoblasts and decreased bone formation. Importantly, inhibition of osteoblast differentiation and new bone formation persists even after complete remission and therefore plays a critical role in the pathogenesis of myeloma bone disease. Several mechanisms involved in the suppression of osteoblasts by myeloma cells have been identified over the years.^16^, ^20^, ^28^, ^90^ Much attention has been drawn to the osteocyte‐derived factor Sclerostin in the recent years.^29^ Circulating Sclerostin levels are increased in postmenopausal women with endocrine responsive breast cancer and in patients with prostate cancer, particularly in individuals receiving androgen deprivation therapy.^93^, ^94^ High levels of SOST/Sclerostin have also been detected in breast cancer cells.^95^ Similarly, elevated Sclerostin expression is found in the circulation of myeloma patients compared with healthy subjects or MGUS patients.^96^, ^97^ Indeed, high SOST mRNA expression was found in plasma cells isolated from small cohorts of patients with myeloma,^98^, ^99^ identifying myeloma/cancer cells as a source of Sclerostin. However, in a study including more than 630 myeloma patients, bone marrow plasma cell SOST mRNA expression was not different compared with healthy controls, nor was it detected in 56 human or murine myeloma cells lines,^100^ suggesting that the elevated serum Sclerostin was driven by another source. Consistent with this notion, the number of osteocytes expressing Sclerostin is higher in bones injected with myeloma cells.^(79,101)^ Moreover, in vitro experiments show that myeloma cells increase SOST mRNA expression in osteocytes, and conditioned media from osteocytes co‐cultured with myeloma cells decreased the expression of Wnt target genes and osteoblast differentiation markers in osteoblastic cells cultured under mineralizing conditions.^79^ As discussed below, genetic and pharmacologic inhibition of Sclerostin increased osteoblast number and induced new bone formation in preclinical models of multiple myeloma bone disease.^97^, ^100^, ^101^ DKK‐1, another Wnt antagonist expressed by osteocytes, is also elevated in the circulation of myeloma patients and breast cancer patients.^102^ In preclinical models, anti‐DKK‐1 successfully prevented myeloma‐induced bone disease and had variable effects on tumor burden leading to clinical trials.^102^, ^103^, ^104^

In osteosarcoma and prostate cancer metastasis, tumor cells secrete factors that stimulate both bone resorption and bone formation. In these cancers, osteoblastic lesions are formed due to increased matrix production by osteoblasts. However, the new bone being deposited is poorly organized, formed primarily of woven tissue.^105^, ^106^ The specific role of the osteocytes as either initiators or drivers of the osteoblastic disease is unclear. Osteocytes, in addition to osteoblasts, may act as a cell of origin for osteosarcoma.^107^ High levels of the osteocyte‐derived factor Sclerostin are also found in prostate cancer patients with osteoblastic bone metastasis.^108^, ^109^ Yet, whether Sclerostin contributes to the osteoblastic disease in these patients remains to be determined.

## Osteocytes and Their Derived Factors as Targets for the Treatment of Cancer That Grows in Bone

Chemotherapeutic agents used in the clinic for the treatment of cancer in bone alter normal bone homeostasis by affecting both osteoblast and osteoclast function. In this section, we will focus on recent results showing that osteocyte function is also affected by chemotherapy, and discuss new findings supporting the potential of targeting osteocytes for the treatment of cancer‐induced bone disease (Fig. 1).

### Antiresorptives

Bisphosphonates are considered the mainstay therapy for cancer patients with bone disease. Bisphosphonates inhibit the activity of osteoclasts and prevent bone loss induced by cancer cells, thus reducing the risk of fracture. The beneficial effects of bisphosphonates on the skeleton are also due to the prevention of osteoblast and osteocyte apoptosis, an effect that depends on the regulation of Connexin 43 (Cx43) signaling between neighboring cells.^110^ Conditioned media from MLO‐Y4 osteocyte cells treated with bisphosphonates reduced the growth, migration, and invasion of MDA‐MB‐231 human breast cancer cells.^111^ These inhibitory effects appear to be mediated by Cx43, as mice with impaired Cx43 gap junctions showed significantly increased tumor burden and an attenuation of the inhibitory effect of bisphosphonates on tumor growth.^111^ This study suggests a role of osteocytes and Cx43 hemichannels in the response to bisphosphonates and identifies Cx43 as a novel therapeutic target.

Denosumab is a fully human monoclonal antibody against RANKL developed as a novel therapeutic agent to inhibit bone resorption.^112^, ^113^ Denosumab is approved to prevent skeletal‐related events in cancer patients with solid tumors (breast and prostate) and bone metastases. In a recent study, Roodman and colleagues found that denosumab has non‐inferiority for the prevention of skeletal‐related events in multiple myeloma patients when compared with zoledronic acid, the mainstay therapy for this patient population. This study also provided clinical evidence of a potential anti‐myeloma effect based on RANKL inhibition.^114^ Based on these promising results, it is expected that denosumab becomes an additional option for treatment of myeloma patients with bone disease. Given that osteocytic RANKL is critical in adult bones and cancer cells increase its expression in osteocytes, it is likely that the potent antiresorptive effects of denosumab on bone are, at least in part, due to the inhibition of osteocyte‐derived RANKL.

### Proteasome inhibitors

Proteasome inhibitors (PIs) prevent the degradation of proteins targeted with poly‐ubiquitin chains.^115^ Treatment with PIs leads to cell cycle arrest and apoptosis in myeloma cancer cells (reviewed in Obeng and colleagues^116^), decreases osteoclast formation and resorption capacity,^117^ and transiently increases bone formation.^117^ In addition to these effects, new actions of PIs have been identified in osteocytes. PI therapy decreased the elevated osteocyte apoptosis found in multiple myeloma patients with bone lesions and prevented the decrease in osteocyte viability induced by co‐culture with myeloma cells in vitro.^92^ Further, myeloma patients receiving the PI bortezomib exhibited a 50% decrease in serum Sclerostin.^96^

### Neutralizing antibodies against Sclerostin

Several neutralizing antibodies against Sclerostin have been developed and have shown remarkable ability to stimulate new bone formation in osteoporotic patients.^118^, ^119^, ^120^, ^121^ Anti‐Sclerostin antibodies stimulate bone gain by enhancing osteoblast function while inhibiting bone resorption. Novel, exciting preclinical data have demonstrated that treatment with anti‐Sclerostin antibodies prevents cancer‐induced bone loss and induces new bone formation in mouse models of multiple myeloma.^97^, ^100^, ^101^ Similarly, anti‐Sclerostin therapy also prevented the bone disease induced by breast cancer cells.^95^ Importantly, anti‐Sclerostin did not affect tumor burden in any of these studies. These promising results provide the rationale for the use of neutralizing antibodies against Sclerostin to stimulate new bone formation and improve bone mass and quality in patients with cancer in bone. Yet, further studies are required to identify the exact source of Sclerostin (osteocytes versus cancer cells) and to determine the effects of anti‐Sclerostin therapy in other bone metastatic cancers. Because of the detection of adverse cardiovascular events in patients treated with anti‐Sclerostin,^122^, ^123^ the FDA has not yet approved the use of anti‐Sclerostin for the treatment of osteoporosis. Of note, a new bispecific antibody targeting both Sclerostin and DKK‐1 has been developed.^124^ However, the performance of this bispecific antibody in humans or cancer is currently unknown.

### Pharmacologic inhibition of Notch signaling

Notch signaling between cancer cells and cells present in the bone/bone marrow microenvironment favors growth and survival of cancer cells and increases bone resorption (reviewed in Colombo and colleagues^82^, ^125^). As mentioned before, physical interactions with myeloma cells activate Notch and induce a rapid programmed cell death (apoptosis) in osteocytes.^79^ In addition, osteocytes reciprocally activate Notch signaling in myeloma cells to stimulate their proliferation.^79^ Thus, targeting the Notch signaling in the metastatic niche could result in multiple beneficial outcomes, including decreasing tumor growth, maintaining osteocyte viability, and inhibiting bone destruction. However, systemic inhibition of Notch signaling using GSIs results in severe gut toxicity, limiting their use in the clinic.^82^, ^125^ Targeting specific components of the Notch pathway has become an attractive approach to avoid the off‐target effects of GSIs. Neutralizing antibodies have been generated to block the Notch ligands Delta‐1 and Jagged‐1^82^, ^125^ and are currently being studied in preclinical models. Another alternative approach to circumvent the side effects of GSIs is to target Notch inhibitors specifically to the bone. Inhibition of Notch signaling using a novel bone‐targeted GSI increased bone mass and decreased bone resorption, without inducing gut toxicity.^126^ However, the effects of this novel agent on tumor growth and cancer‐induced bone disease remain to be determined. Future research efforts are needed to define the specific effects of Notch signaling in osteocytes and to determine the effectiveness of targeting the Notch pathway to treat cancer in bone.

## Conclusion and Future Directions

Recent studies have identified osteocytes as important components of the cancer microenvironment in bone. We now have evidence that cancer cells alter osteocyte viability and their gene expression profile. These changes transform osteocytes into pro‐tumorigenic cells and enhance their osteoclastogenic potential, leading to tumor growth and bone destruction. In addition, through the overproduction of Wnt antagonists, osteocytes also contribute to the suppression of bone formation. However, much work remains to be done to understand the full involvement of osteocytes in cancer metastases to bone. For instance, the role of osteocytes in the early steps of bone metastasis is practically unknown. Moreover, the mechanisms underlying osteocyte‐tumor cell interactions and the consequences for bone homeostasis are not fully understood. Further, most of the available data derive from myeloma and breast cancer patients and mouse models. Thus, whether osteocytes play a similar role in other metastatic cancers remains to be determined.

Although we have just begun to scratch the surface regarding the role of osteocytes in cancer, targeting osteocytic pathways and molecular messengers has already proven to be successful to prevent/improve the associated bone disease. These promising results support that targeting osteocytes in the cancer niche has the potential to guide the development of new therapeutic regimens for the treatment of cancer in bone. In conclusion, osteocytes contribute to the progression of cancer in bone. Thus, full understanding of the mechanisms by which osteocytes influence cancer cells, osteoblasts, and osteoclasts should increase the repertoire of pharmacological approaches to combat cancer in bone.

## Disclosures

Authors have no conflicts of interest to report.
